# Risk of Pre‐Stroke Malnutrition Predicts Mortality in Ischaemic Stroke Patients Undergoing Thrombectomy

**DOI:** 10.1111/jhn.70181

**Published:** 2025-12-17

**Authors:** Ghil Schwarz, Angelo Cascio Rizzo, Guglielmo Carlo Pero, Francesca Poggetti, Andrea Magi, Simone Bellavia, Antonella Gatti, Mariangela Piano, Elio Clemente Agostoni, Maria Sessa

**Affiliations:** ^1^ Department of Neurology and Stroke Unit ASST Grande Ospedale Metropolitano Niguarda Milan Italy; ^2^ Department of Neuroradiology ASST Grande Ospedale Metropolitano Niguarda Milan Italy

## Abstract

**Background and Aims:**

Malnutrition is common in older adults but poorly studied in acute ischaemic stroke (AIS) patients treated with endovascular thrombectomy (EVT). This study assesses the prevalence of malnutrition risk at Stroke Unit admission, related clinical factors, and its impact on EVT outcomes, functional status and 90‐day mortality.

**Methods:**

Retrospective single‐centre study on consecutive EVT‐treated AIS patients (2021–2024). Malnutrition risk was assessed using the GNRI ([1.489 × serum albumin g/L] + [41.7 × current/ideal body weight]). For the primary analyses, patients with GNRI ≤ 98 were classified as at risk of malnutrition (RoM+); secondary analyses further stratified moderate‐to‐severe risk (GNRI < 92). Logistic regression was used to identify factors associated with RoM+ and to evaluate its impact on (1) EVT performance measures, (2) 90‐day mRS and (3) 90‐day mortality. Multivariable models included variables with *p* < 0.10 in univariate testing, and the robustness of outcome analyses was assessed through sensitivity models including predefined clinically relevant covariates.

**Results:**

Among 306 patients, 43.8% were classified as RoM+, including 24.5% with moderate‐to‐severe nutritional risk. In the multivariable model, RoM+ was independently associated with older age (aOR 1.03, 95% CI 1.00–1.06), higher pre‐stroke disability (aOR 1.48, 95% CI 1.12–1.95) and a lower prevalence of dyslipidemia (aOR 0.59, 95% CI 0.35–0.97) and was not associated with EVT efficacy or safety. RoM+ was independently associated with increased 90‐day mortality (aOR 2.37, 95% CI 1.06–5.28), while the association with functional outcome did not reach statistical significance (aOR 1.58, 95% CI 0.98–2.55). Secondary analyses using GNRI severity strata and predefined sensitivity models showed consistent results.

**Conclusion:**

At admission, ~44% of EVT‐treated AIS patients are at risk of malnutrition, which independently predicted 90‐day mortality. Pre‐stroke nutritional status may represent a modifiable prognostic factor in stroke care and warrants further prospective investigation.

## Summary

- Risk of malnutrition (RoM), assessed using the Geriatric Nutritional Risk Index (GNRI) at Stroke Unit admission, remains underinvestigated in acute ischaemic stroke (AIS), particularly in patients undergoing endovascular thrombectomy (EVT).
- ~44% of EVT‐treated AIS patients met criteria for RoM (GNRI ≤ 98), which was associated with older age, greater pre‐stroke disability and a lower prevalence of dyslipidemia.
- RoM independently predicted 90‐day mortality, with a stronger association observed in patients with moderate‐to‐severe risk (GNRI < 92).
- Incorporating pre‐stroke RoM screening into early clinical evaluation—alongside routine post‐stroke dysphagia assessment—may help identify a potentially modifiable prognostic factor.

## 1 Introduction

Malnutrition is a complex clinical condition that includes both undernutrition and overnutrition, reflecting an imbalance between nutrient intake and the body's requirements [1]. According to the European Society for Clinical Nutrition and Metabolism (ESPEN), undernutrition is characterized by reduced nutrient intake or absorption, leading to altered body composition—specifically a reduction in lean body mass—and impairments in both physical and mental function [2]. In this study, we will use the term malnutrition as synonymous with undernutrition.

The prevalence of malnutrition in healthcare settings is particularly concerning, affecting approximately 29% of patients [3]. Among elderly populations, the prevalence is even higher, reaching rates as high as 70% [4]. This population is especially vulnerable due to various factors, including muscle mass loss, dysphagia, reduced nutrient absorption related to chronic diseases and inflammation, cognitive decline and dementia and social isolation. The clinical manifestations of malnutrition include unintentional weight loss, reduced muscle mass and strength, osteoporosis with an increased risk of fractures, anaemia and neuropathies. These are independently associated with adverse outcomes such as prolonged hospital stays, increased mortality and heightened susceptibility to infections [5].

Malnutrition is a critical yet underexplored aspect of acute ischaemic stroke (AIS), despite its high prevalence among older individuals [6]. Current guidelines emphasize dysphagia screening in all patients with AIS [7], focusing on malnutrition as a consequence of the stroke. However, few studies have addressed nutritional status at the time of stroke onset, prior to hospital‐related factors.

In particular, data on pre‐stroke malnutrition risk in patients undergoing endovascular treatment (EVT) for AIS are scarce. Most existing studies are based on Asian populations or include heterogeneous stroke types and phases, with little focus on EVT‐treated patients. Moreover, the use of different assessment tools limits comparability [6]. Among available tools, the Geriatric Nutritional Risk Index (GNRI) is a practical and validated method for estimating malnutrition risk, especially in elderly individuals [8].

This study aims to investigate (1) the prevalence of malnutrition risk at the time of Stroke Unit admission in patients with AIS undergoing EVT, (2) the demographic and clinical characteristics associated with this condition, (3) its impact on EVT efficacy and safety and (4) its influence on 90‐day functional outcomes and mortality.

## 2 Methods

This is a single‐centre, retrospective study that included patients with AIS who underwent EVT and were consecutively admitted to the Stroke Unit at Niguarda Hospital between 1 January 2021 and 30 April 2024. An additional inclusion criterion was the availability of the variables required to calculate the GNRI score [8].

Based on the Stroke Unit database of Niguarda Hospital, we collected data across several categories. Demographic variables and risk factors included age, sex, ethnicity, body mass index (BMI = weight [kg]/height [m^2^]) and smoking status. Relevant medical history, such as previous ischaemic stroke, pre‐morbid modified Rankin Scale (mRS), arterial hypertension, diabetes, dyslipidemia and atrial fibrillation, was also recorded.

Acute assessment variables encompassed both clinical and imaging data. Clinical parameters included baseline NIH Stroke Scale (NIHSS) score, Mean Arterial Pressure (MAP = [2/3 × diastolic blood pressure] + [1/3 × systolic blood pressure]) and blood glucose levels. Imaging assessments included Alberta Stroke Program Early CT Score (ASPECTS [9]) or posterior circulation ASPECTS (pc‐ASPECTS [10]) and collateral status, which was evaluated separately for anterior circulation [11]. (classified as “poor” [0–3] or “good” [4–5]) and posterior circulation [12]. The type of vascular occlusion was categorized as large vessel occlusion (LVO) or medium vessel occlusion (MeVO), with anterior circulation involvement and intravenous thrombolysis (IVT) also documented. LVOs were defined as occlusions in major arteries, such as the internal carotid artery, M1 segment of the middle cerebral artery, M2 dominant or co‐dominant branches, vertebral artery and basilar artery. MeVOs included occlusions in the A1, A2 and A3 segments of the anterior cerebral artery, M2 non‐dominant branches, M3 segments of the middle cerebral artery, and P1, P2 and P3 segments of the posterior cerebral artery [13]. For patients presenting with multiple occlusions, the most proximal occlusion was used for classification.

EVT‐related efficacy and safety variables included: procedural times, such as onset‐to‐groin (OTG) and onset‐to‐reperfusion (OTR), as well as technical metrics, like the number of EVT passes, successful reperfusion (defined as mTICI ≥ 2b [14]) and the presence of haemorrhagic transformation. Haemorrhagic complications included intracerebral haemorrhage (ICH [15]) or subarachnoid haemorrhage (SAH), detected on the first post‐procedure CT scan and confirmed by a follow‐up CT performed at least 48 h later. Symptomatic intracerebral haemorrhage (sICH) was defined as the presence of any haemorrhagic transformation detected on neuroimaging at the 24/36‐h assessment accompanied by a simultaneous increase of ≥ 4 points on the NIHSS compared with the pre‐EVT clinical evaluation [16]. EVT‐related complications, such as vessel perforation, arterial dissection, embolization into a new vascular territory, arterial access complications and early reocclusion, were also recorded [17].

### 2.1 GNRI Score

The GNRI was calculated using the formula: GNRI = (1.489 × serum albumin in g/L) + (41.7 × current weight in kg/ideal weight in kg). The ideal weight was determined using the Lorentz formula: for men, ideal weight (kg) = Height in cm − 100 − ([Height in cm − 150]/4); for women, ideal weight (kg) = Height in cm − 100 − ([Height in cm − 150]/2.5). In line with the original GNRI publication, the GNRI was used to screen for nutritional risk, not to diagnose malnutrition [8]. Patients with a GNRI > 98 were classified as not at risk of malnutrition (RoM−), while those with a GNRI ≤ 98 were considered at risk of malnutrition (RoM+). To further explore potential gradient effects, the RoM+ group was additionally stratified according to established GNRI risk categories: GNRI 92–98 (mild risk), 82–92 (moderate risk) and < 82 (severe risk). For inferential analyses, the moderate and severe categories were subsequently combined (GNRI < 92) to ensure adequate subgroup size and maintain statistical interpretability. GNRI scores were calculated retrospectively for research purposes and did not influence patient management. Serum albumin levels required for GNRI calculation were obtained during routine blood draws on the first day after admission. Anthropometric measures (weight and height) were initially obtained from clinical history at admission through direct questioning of the patient or caregiver. The reported values were subsequently cross‐validated by the clinical team using bedside observation and estimation. Final values recorded in the medical chart—and used for analysis—were derived from this integrated assessment process.

### 2.2 Statistical Analyses

Descriptive statistics were used to characterize the study population and determine the prevalence of nutritional risk. Categorical variables were reported as counts and proportions, while continuous variables were summarized as medians with interquartile ranges (IQR). The prevalence of RoM+ was also analysed across different age groups (< 60 years, 60–80 years and > 80 years).

To identify pre‐EVT variables associated with RoM+, a univariate analysis was first performed using the chi‐squared test (or Fisher's exact test, when appropriate) for categorical variables and the Wilcoxon rank‐sum test for continuous variables. Variables with a *p*‐value < 0.1 in the univariate analysis were subsequently included in a multivariate logistic regression to identify pre‐treatment factors independently associated with RoM+. Given the expected collinearity between BMI and GNRI (due to shared weight dependence), BMI was not included in the multivariable models. The same approach was applied to EVT‐related efficacy and safety variables.

For outcome analysis, both (1) RoM+ status and the subgroup with (2) moderate‐to‐severe nutritional risk were evaluated. First, univariate associations between these GNRI‐based categories and the two predefined clinical endpoints—90‐day mRS (analysed as an ordinal variable for shift analysis) and 90‐day mortality (binary)—were assessed. Follow‐up at 90 days was obtained primarily through scheduled outpatient visits; when this was not feasible, structured telephone interviews with the patient or caregiver were conducted. Variables showing a *p*‐value < 0.1 in their univariate association with each outcome were subsequently entered into separate multivariable models to evaluate the independent prognostic effect of nutritional status on functional outcome and mortality. The results of these multivariate analyses were graphically represented using forest plots. To ensure a clinically informed model specification and avoid outcome‐dependent variable selection, multivariable analyses were additionally repeated using a predefined set of prognostically relevant covariates, irrespective of their univariate statistical association. These included age, pre‐stroke mRS, diabetes mellitus and admission blood glucose, baseline NIHSS, ASPECTS, collateral status, anterior versus posterior circulation occlusion, intravenous thrombolysis, onset‐to‐recanalization time, successful reperfusion (mTICI ≥ 2b), EVT‐related complications and symptomatic intracranial haemorrhage.

A univariate analysis was also conducted to compare the baseline characteristics of included *versus* excluded patients as part of a selection bias analysis.

Statistical significance was set at a threshold of *p* < 0.05, and all analyses were conducted using STATA software (version 18, StataCorp, College Station, TX, USA).

The study adhered to the Strengthening the Reporting of Observational Studies in Epidemiology (STROBE) guidelines [18].

## 3 Results

A total of 364 patients underwent EVT during the study period, of whom 306 (84.1%) had all the necessary variables for GNRI score calculation and were therefore included in the analysis. Patients excluded due to missing GNRI score data (Supporting Information S3: Table S1) were older (80 vs. 75 years, *p* = 0.019), had higher pre‐morbid mRS (*p* = 0.002) and showed worse outcomes, including higher 90‐day mortality (27.6% vs. 14.7%, *p* = 0.016).

The baseline characteristics of the included population are presented in Table 1. The median age was 75 years (IQR 64–82), with 54.9% of patients being female. The median NIHSS score at admission was 13 (IQR 8–18), and 31.7% of patients received IVT prior to EVT. Based on GNRI, 134 patients (43.8%) were classified as RoM+ and 172 (56.2%) as RoM−. Among RoM+ patients, 59 (44.0%) were at mild risk (GNRI 92–98), 53 (39.6%) at moderate risk (GNRI 82–92) and 22 (16.4%) at severe risk (GNRI < 82). The prevalence of RoM+ increased significantly with age, rising from 35% (14/40) in patients under 60 years and 36% (59/162) in patients aged 60–80 years to 58.7% in those over 80 years.

**Table 1:** Descriptive statistics and univariate analysis of demographic, clinical and acute variables in the entire cohort and by risk of malnutrition.

	Entire cohort *N* = 306	Risk of malnutrition + 134/306 (43.8%)	Risk of malnutrition‐172/306 (56.2%)	*p* value
Baseline variables				
Age (median [IQR])	75 [64–82] (306)	79 [69–85]	71 [62–79]	< 0.001
Female sex	168/306 (54.9%)	77/134 (57.5%)	92/172 (52.9%)	0.428
Caucasian ethnicity	298/306 (97.4%)	132/134 (98.5%)	166/172 (96.5%)	0.462
BMI (median [IQR])	25.7 [23.8–28.1] (306)	23.7 [21.4–25.6]	27.6 [25.4–30.1]	< 0.001
Active smoking	54/306 (17.6%)	27/134 (20.1%)	27/172 (15.7%)	0.311
Previous ischaemic stroke	32/306 (10.5%)	10/134 (7.5%)	22/172 (12.8%)	0.131
Pre‐morbid mRS (median [IQR])	0.0 [0.0–1.0] (306)	1 [0–2]	0 [0–1]	< 0.001
Arterial hypertension	221/306 (72.2%)	94/134 (70.1%)	127/172 (73.8%)	0.475
Diabetes mellitus	46/306 (15.0%)	16/134 (11.9%)	30/172 (17.4%)	0.182
Dyslipidemia	105/306 (34.3%)	39/134 (29.1%)	66/172 (38.4%)	0.090
Atrial fibrillation	85/306 (27.8%)	43/134 (32.1%)	42/172 (24.4%)	0.137
Acute assessment				
NIHSS (median [IQR])	13.0 [8.0–18.0] (306)	12 [8–17]	14 [8–18]	0.341
MAP (median [IQR])	105 [93–120] (288)	103 [92–117] (125)	105 [95–120] (163)	0.435
Blood glucose (median [IQR])	121 [104–148] (297)	118 [103–148] (130)	122 [106–148] (167)	0.806
ASPECTS/pc‐ASPECTS (median [IQR])	9.0 [8.0–10.0] (299)	9 [8–10] (131)	9 [8–10] (168)	0.570
Good collateral status	215/306 (70.3%)	90/134 (67.2%)	125/172 (72.7%)	0.295
LVO (vs. MeVO)	264/306 (86.3%)	119/134 (88.8%)	145/172 (84.3%)	0.256
Anterior circulation	281/306 (91.8%)	124/134 (92.5%)	157/172 (91.3%)	0.690
IVT	97/306 (31.7%)	39/134 (29.1%)	58/172 (33.7%)	0.389
EVT‐related variables				
OTG (median [IQR])	271.0 [185.0–633.0] (306)	280 [188–684]	262 [181–560]	0.306
Number of EVT passes (median [IQR])	1 [1−2] (306)	1 [1−2] (134)	1 [1−2] (172)	0.674
OTR (median [IQR])	332.5 [224.0–682.0] (306)	374 [238–723]	315 [217–627]	0.252
EVT complications	28/306 (9.2%)	11/134 (8.2%)	17/172 (9.9%)	0.614
mTICI ≥ 2b	276/306 (90.2%)	121/134 (90.3%)	155/172 (90.1%)	0.958
Any haemorrhagic transformation	45/306 (14.7%)	16/134 (11.9%)	29/172 (16.9%)	0.228
Symptomatic haemorrhagic transformation	9/306 (2.9%)	5/134 (3.7%)	4/172 (2.3%)	0.470
90‐day outcomes				
mRS at 90‐day (median [IQR])	2 (0–4) [306]	3 (1–5)	2 (0–4)	0.001
mRS 0–2 at 90‐day	166/306 (54.8%)	61/134 (45.4%)	105/172 (61.1%)	0.007
Death at 90‐day	45/306 (14.7%)	28/134 (20.9%)	17/172 (9.9%)	0.007

Abbreviations: ASPECTS, Alberta Stroke Program Early CT Score; BMI, body mass index; GNRI, Geriatric Nutritional Risk Index; IVT, intravenous thrombolysis; LVO, large vessel occlusion; MAP, mean arterial pressure; MeVO, medium vessel occlusion; mRS, Modified Rankin Scale; mTICI, modified thrombolysis in cerebral infarction; NIHSS, National Institutes of Health Stroke Scale; OTG, onset‐to‐groin time; OTR, onset‐to‐reperfusion time.

In the univariate analysis, several pre‐EVT variables were significantly associated with RoM+ status (Table 1). In the multivariate model (Table 2), RoM+ was independently associated with older age (aOR 1.03, 95% CI 1.00–1.06, *p* = 0.016), higher pre‐morbid mRS (aOR 1.48, 95% CI 1.12–1.95, *p* = 0.005) and a lower prevalence of dyslipidemia (aOR 0.59, 95% CI 0.35–0.97, *p* = 0.039).

**Table 2:** Multivariate logistic regression analysis for predictors of risk of malnutrition in ischaemic stroke patients undergoing EVT.

	Malnutrition aOR (95% CI)	*p* value
Age	1.03 (1.00−1.065)	0.016
Pre‐morbid mRS	1.48 (1.12−1.95)	0.005
Dyslipidemia	0.59 (0.35−0.97)	0.039

Abbreviations: aOR, adjusted odds ratio; mRS, Modified Rankin Scale.

No significant differences in EVT‐related efficacy or safety variables were observed between RoM+ and RoM− patients in the univariate analysis. Therefore, no multivariate model was applied for EVT‐related outcomes.

Several variables, including GNRI, were associated with both 90‐day mRS and 90‐day mortality in the univariate analysis (Supporting Information S3: Tables S2 and S3). RoM+ patients had worse 90‐day functional outcomes (OR 1.96, 95% CI 1.31–2.93, *p* = 0.001) and higher 90‐day mortality (OR 2.41, 95% CI 1.26–4.62, *p* = 0.008). A graded effect was also observed when considering GNRI severity strata, with patients in the moderate‐to‐severe risk category showing higher odds of poor functional outcome (OR 2.21, 95% CI 1.36–3.57, *p* = 0.001) and mortality (OR 2.88, 95% CI 1.39–5.97, *p* = 0.004) compared with those without malnutrition risk.

In the multivariate analysis for 90‐day mRS (Supporting Information S1: Figure S1), significant predictors included age (aOR 1.06, 95% CI 1.03–1.08, *p* < 0.001), pre‐morbid mRS (aOR 2.07, 95% CI 1.56–2.73, *p* < 0.001), baseline NIHSS (aOR 1.14, 95% CI 1.10–1.19, *p* < 0.001), ASPECTS (aOR 0.71, 95% CI 0.58–0.86, *p* = 0.001), and the number of EVT passes (aOR 1.32, 95% CI 1.08–1.62, *p* = 0.007). RoM+ was no longer significantly associated with functional outcome after adjustment (aOR 1.58, 95% CI 0.98–2.55, *p* = 0.059). Consistently, the moderate‐to‐severe GNRI subgroup showed a similar nonsignificant trend (aOR 1.57, 95% CI 0.88–2.82, *p* = 0.128) (Table 3).

**Table 3:** Association of risk of malnutrition (GNRI ≤ 98) and moderate/severe risk of malnutrition (GNRI < 92) with 90‐day functional outcome and mortality.

	90‐day mRS				90‐day mortality			
	OR (95% CI)	*p* value	aOR (95% CI)^a^	*p* value	OR (95% CI)	*p* value	aOR (95% CI)^b^	*p* value
Risk of malnutrition+	1.96 (1.31−2.93)	0.001	1.58 (0.98−2.55)	0.059	2.41 (1.26−4.62)	0.008	2.37 (1.06−5.28)	0.035
Moderate/Severe risk of malnutrition	2.21 (1.36−3.57)	0.001	1.57 (0.88−2.82)	0.128	2.88 (1.39−5.97)	0.004	2.96 (1.16−7.55)	0.023

a Adjusted for variables associated with the outcome at *p* < 0.10: age, sex, pre‐stroke mRS, arterial hypertension, diabetes mellitus, atrial fibrillation, baseline NIHSS, mean arterial pressure, blood glucose, ASPECTS, collateral status, onset‐to‐groin time, number of EVT passes, EVT‐related complications, successful reperfusion (mTICI ≥ 2b) and any intracranial haemorrhage.

^b^ Adjusted for variables associated with the outcome at *p* < 0.10: age, pre‐stroke mRS, atrial fibrillation, baseline NIHSS, blood glucose, ASPECTS, collateral status, intravenous thrombolysis, number of EVT passes and any intracranial haemorrhage.

For 90‐day mortality (Supporting Information S2: Figure S2), significant predictors included age (aOR 1.04, 95% CI 1.00–1.09, *p* = 0.050), baseline NIHSS (aOR 1.07, 95% CI 1.00–1.14, *p* = 0.046), and number of EVT passes (aOR 1.35, 95% CI 1.03–1.76, *p* = 0.029). Importantly, RoM+ remained independently associated with increased mortality (aOR 2.37, 95% CI 1.06–5.28, *p* = 0.035). The association was even more pronounced when restricting the analysis to patients with moderate‐to‐severe nutritional risk (aOR 2.96, 95% CI 1.16–7.55, *p* = 0.023) (Table 3).

In sensitivity analysis using a prespecified adjustment set, findings remained consistent with the primary models. RoM+ continued to show a similar effect size for functional outcome without reaching statistical significance (aOR 1.56, 95% CI 0.98–2.46, *p* = 0.059), while the association with mortality remained significant (aOR 2.29, 95% CI 1.03–5.10, *p* = 0.042) (Supporting Information S3: Table S4).

## 4 Discussion

This single‐centre retrospective study on consecutive ischaemic stroke patients undergoing mechanical thrombectomy demonstrates that being at risk of malnutrition at the time of Stroke Unit admission (1) is highly prevalent, (2) is associated with advanced age, greater pre‐stroke disability and lower prevalence of dyslipidemia, (3) does not affect the procedural efficacy or safety of endovascular treatment and (4) is independently associated with increased 90‐day mortality, especially in patients with moderate‐to‐severe nutritional risk.

The role of nutrition in ischaemic stroke is a key focus following the acute event, with national and international guidelines recommending prompt dysphagia screening for all hospitalized patients [7]. The primary aim is to prevent aspiration and reduce the risk of malnutrition during hospitalization. However, pre‐existing vulnerability before stroke onset remains poorly explored. To our knowledge, this is the first Western study investigating pre‐stroke malnutrition risk in a homogeneous cohort of EVT‐treated patients, revealing a prevalence of RoM+ close to 45%. This proportion rises to nearly 60% among patients over 80, highlighting the strong association between poor baseline nutritional status and advanced age [19]. Malnutrition risk in older adults results from a multifactorial interplay of social, clinical and physiological determinants. Factors such as social isolation, financial hardship and poor access to quality food may reduce intake [20], while dysphagia, depression and chronic illness further impair consumption [21, 22]. Age‐related physiological changes—reduced appetite, sensory decline, delayed gastric emptying, altered hormonal signals—also play a key role [23, 24]. Sarcopenia and cachexia, common in older frail patients, further worsen nutritional reserves [25, 26]. Overall, our data reinforce the role of age and functional dependence as independent correlates of RoM+. Notably, RoM+ was associated with a lower prevalence of dyslipidemia. This finding should be interpreted with caution, as dyslipidemia in our dataset was recorded as a medical history variable rather than based on measured lipid levels. Nonetheless, the association is consistent with the “reverse epidemiology” described in frail and sarcopenic older adults, where lower lipid values may reflect chronic undernutrition and catabolic status rather than a healthier metabolic profile [27].

Our findings show that being at risk of malnutrition is independently associated with higher 90‐day mortality but not with functional outcome. In our cohort, predictors of worse functional outcome were age, pre‐stroke disability, baseline NIHSS, ASPECTS and the number of EVT passes—patterns fully consistent with established determinants of post‐thrombectomy prognosis [28]. Likewise, predictors of mortality included age, baseline stroke severity and the number of EVT attempts, aligning with prior evidence in comparable populations [28]. The absence of an association between poor outcomes and sICH in our dataset is most likely explained by the low event rate rather than a true lack of clinical relevance. Within this expected prognostic framework, RoM+ emerged as an independent predictor of 90‐day death. Notably, the association was stronger among patients with moderate‐to‐severe nutritional risk, suggesting a potential dose–response effect. Furthermore, the findings remained consistent in sensitivity analyses using a predefined clinically driven covariate set, strengthening confidence in the robustness and generalizability of the observed association. Although malnutrition has previously been linked to adverse outcomes in ischaemic stroke,⁶ this study is the first to demonstrate its prognostic relevance in a strictly EVT‐treated Western population. These results highlight pre‐stroke nutritional vulnerability as a frequently overlooked yet potentially modifiable determinant of outcome. The mechanisms linking malnutrition and mortality are likely multifactorial. Impaired nutritional status weakens immune function, increasing susceptibility to infections such as pneumonia and urinary tract infections, both common and severe in stroke patients. In addition, sarcopenia—frequently associated with malnutrition—may impair respiratory capacity and functional recovery, worsening prognosis. These considerations support the role of baseline nutritional status as a modifiable risk factor. Notably, no association was found between RoM+ and EVT technical outcomes. Patients classified as RoM+ achieved similar reperfusion rates and complication profiles as RoM− counterparts. This suggests that the clinical impact of RoM+ may emerge primarily during the subacute or recovery phase, rather than during the procedure itself. The longer median procedural times observed in RoM+ patients (94 vs. 53 min) may reflect anatomical factors, such as vessel tortuosity, which is more frequent in older, frail individuals.

This study has several limitations. It is retrospective and single‐centre, with potential selection bias: patients lacking GNRI data were generally older, more disabled and had higher mortality—likely due to early clinical deterioration precluding albumin measurement. Our findings thus reflect patients who survived beyond the hyperacute phase. Another limitation is the use of GNRI as the sole assessment tool. While it is validated and widely used, GNRI remains a screening index, not a diagnostic instrument. According to ESPEN guidelines [2], a formal malnutrition diagnosis requires further clinical evaluation. Our study identifies patients *at risk* rather than those with *confirmed* malnutrition, a distinction that must be considered when interpreting the results. Future prospective studies should include comprehensive nutritional assessments. Furthermore, the GNRI, by integrating serum albumin and body weight, may partly reflect sarcopenia rather than pure malnutrition, particularly in older patients with reduced muscle mass. Sarcopenia and malnutrition share overlapping pathophysiological mechanisms, including inflammation, hormonal dysregulation and loss of lean body mass, all of which contribute to frailty and poorer outcomes after stroke [29, 30]. Although direct measures of muscle mass or strength were not available in our dataset, this overlap should be considered when interpreting the association between low GNRI and mortality, which may partly capture the prognostic effect of sarcopenia‐related vulnerability. Future studies should therefore combine GNRI with specific assessments of muscle mass or function—such as bioelectrical impedance, handgrip strength or CT‐based muscle quantification—to better distinguish the respective roles of malnutrition and sarcopenia in post‐stroke prognosis. Moreover, we lacked detailed data on social, dietary and functional contributors to malnutrition risk—such as weight loss, food intake, medications or chronic gastrointestinal disease. In addition, we did not collect specific data on post‐stroke dysphagia, which may have influenced nutritional status during the early phase of hospitalization. The absence of direct anthropometric measurements may have introduced bias in weight and height estimation and affected the accuracy of GNRI calculation. Nonetheless, this scenario closely mirrors the context of most Stroke Units, where direct measurement is difficult to perform in everyday clinical settings. Finally, although albumin was collected early (on the first available working day after admission), it may still reflect acute‐phase responses.

## 5 Conclusion

Pre‐stroke malnutrition risk was common in AIS patients undergoing EVT and independently associated with 90‐day mortality. These findings support recognizing pre‐stroke nutritional status as a potentially modifiable risk factor, deserving early screening and targeted interventions—alongside established practices for managing post‐stroke dysphagia.

## Author Contributions

Ghil Schwarz led the study conception, design, data analysis, interpretation and manuscript drafting. Angelo Cascio Rizzo contributed substantially to study methodology, data interpretation and manuscript development. Guglielmo Carlo Pero, Francesca Poggetti, Andrea Magi, Simone Bellavia, Antonella Gatti and Mariangela Piano contributed to data collection and critically revised the manuscript for important intellectual content. Elio Clemente Agostoni contributed to clinical oversight and manuscript review. Maria Sessa provided senior supervision and critical revision of the final manuscript. All authors approved the final version and agree to be accountable for all aspects of the work.

## Funding

The authors received no specific funding for this work.

## Ethics Statement

This study was conducted in accordance with institutional and national regulations governing research ethics. Because the study used fully anonymized retrospective clinical data and involved no intervention or modification of clinical care, formal Institutional Review Board (IRB) approval and informed consent requirements were waived. At hospital admission, patients were routinely informed that their clinical data might be used for research purposes, and written consent was obtained when possible. For individuals unable to provide consent and without a legal representative, anonymized data collected during routine clinical care were used in accordance with institutional policy and ethics committee guidance. All procedures performed in studies involving human participants were in accordance with the ethical standards of the institutional and/or national research committee and with the 1964 Helsinki declaration and its later amendments.

## Conflicts of Interest

The authors declare no conflicts of interest.

## Supporting information


**Supporting Figure 1:** Multivariate Analysis of Factors Associated with 90‐Day Functional Outcome (mRS). Forest plot illustrating the multivariate analysis of variables associated with 90‐day modified Rankin Scale (mRS) outcomes. Adjusted odds ratios (aOR) and 95% confidence intervals (CI) are displayed for each variable, derived from a single logistic regression model that included all selected predictors. The dashed vertical line at aOR = 1 indicates no effect. Variables with p‐values < 0.05 are considered statistically significant.

**Supporting Figure 2:** Multivariate Analysis of Factors Associated with 90‐Day Mortality. Forest plot depicting the multivariate analysis of factors associated with 90‐day mortality. Each variable's adjusted odds ratio (aOR) and 95% confidence interval (CI) are reported, based on a multivariable logistic regression model. The vertical dashed line at aOR = 1 represents no association with mortality. Statistical significance is defined as p < 0.05.

Malnutrition Supplementary Clean.
