# Supplementary figures and images for: Risk of Pre‐Stroke Malnutrition Predicts Mortality in Ischaemic Stroke Patients Undergoing Thrombectomy
Source: J Hum Nutr Diet. 2025 Dec 17;38(6):e70181. doi: 10.1111/jhn.70181 (PMC12712263; doi:10.1111/jhn.70181)

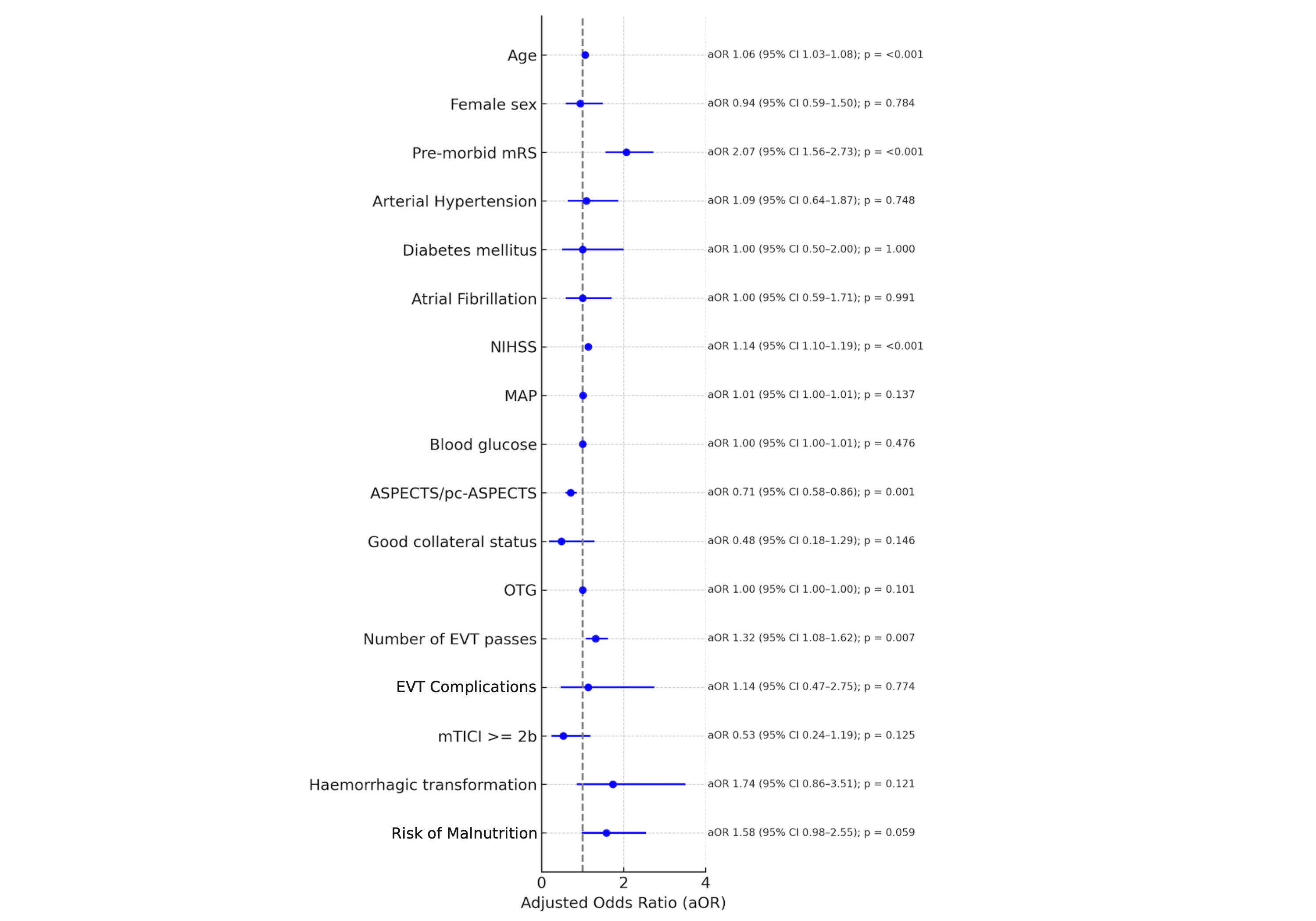

Supplement: Supplementary file 1 — Supporting Figure 1: Multivariate Analysis of Factors Associated with 90‐Day Functional Outcome (mRS). Forest plot illustrating the multivariate analysis of variables associated with 90‐day modified Rankin Scale (mRS) outcomes. Adjusted odds ratios (aOR) and 95% confidence intervals (CI) are displayed for each variable, derived from a single logistic regression model that included all selected predictors. The dashed vertical line at aOR = 1 indicates no effect. Variables with p‐values < 0.05 are considered statistically significant. [file JHN-38-0-s002.png]

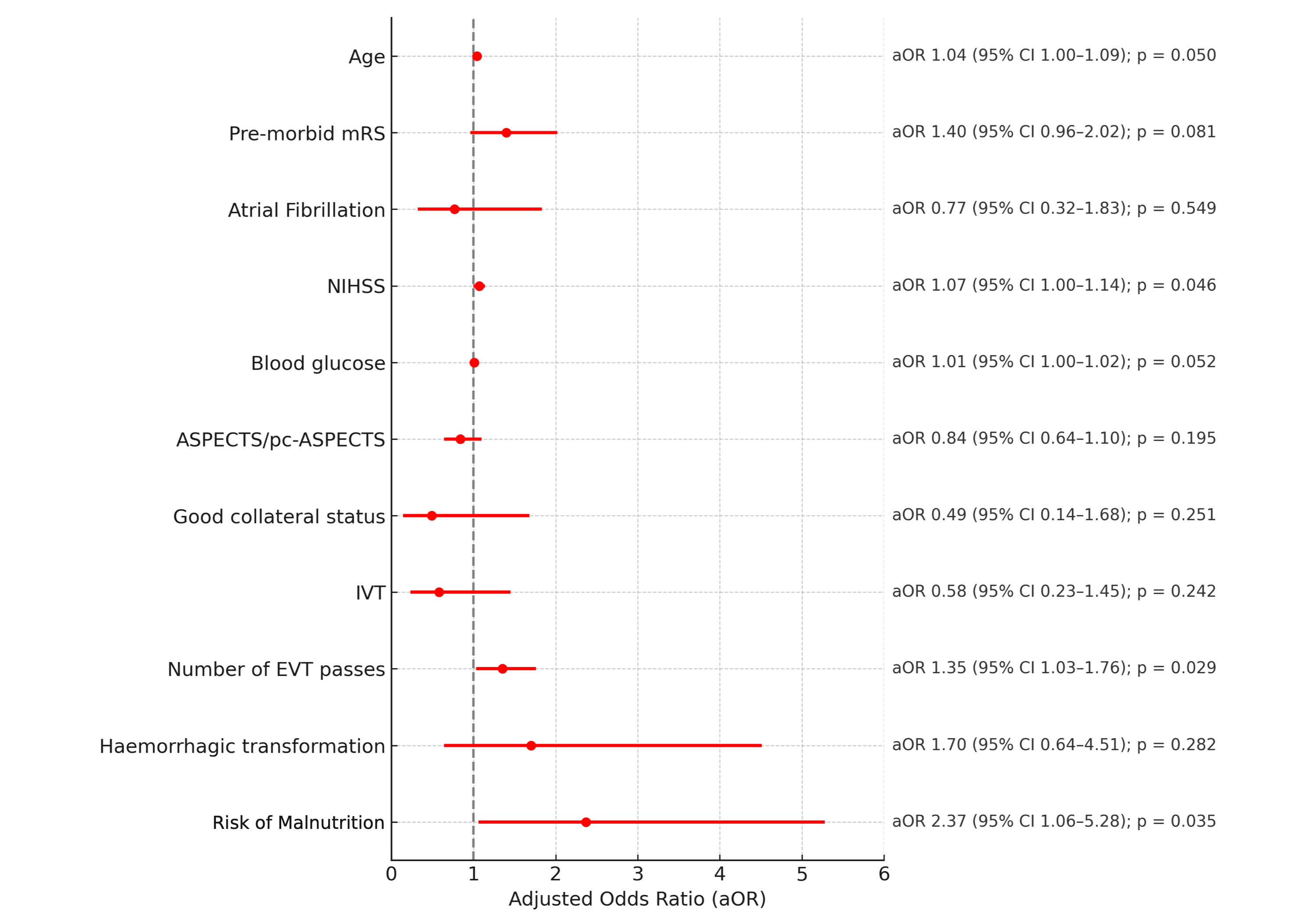

Supplement: Supplementary file 2 — Supporting Figure 2: Multivariate Analysis of Factors Associated with 90‐Day Mortality. Forest plot depicting the multivariate analysis of factors associated with 90‐day mortality. Each variable's adjusted odds ratio (aOR) and 95% confidence interval (CI) are reported, based on a multivariable logistic regression model. The vertical dashed line at aOR = 1 represents no association with mortality. Statistical significance is defined as p < 0.05. [file JHN-38-0-s003.png]
