# Supplementary material for: Risk of Pre‐Stroke Malnutrition Predicts Mortality in Ischaemic Stroke Patients Undergoing Thrombectomy
Source: J Hum Nutr Diet. 2025 Dec 17;38(6):e70181. doi: 10.1111/jhn.70181 (PMC12712263; doi:10.1111/jhn.70181)
Supplement: Supplementary file 3 — Malnutrition Supplementary Clean. [file JHN-38-0-s001.docx]

Supplementary to:

**Risk of Pre-Stroke Malnutrition Predicts Mortality in Ischemic Stroke Patients Undergoing Thrombectomy**

| **Supplementary Table S1.** Selection bias analysis showing the differences between patients included in the analyses and those excluded (due to the lack of necessary data to calculate the GNRI score). | | | |
| --- | --- | --- | --- |
|  | **Excluded**  **N=58** | **Included**  **N=306** | p-value |
| **Demographics variables** |  |  |  |
| Age (median [IQR]) | 80 (66 - 85) | 75 (64 - 82) | 0.019 |
| Male gender | 23/58 (39.7%) | 138/306 (45.1%) | 0.444 |
| Caucasian ethnicity | 55/58 (94.8%) | 298/306 (97.4%) | 0.297 |
| BMI (median [IQR]) | 25.3 (23.9 - 25.8) | 25.7 (23.8 - 28.1) | 0.199 |
| **Medical history and risk factors** |  |  |  |
| Active smoking | 4/58 (6.9%) | 54/306 (17.6%) | 0.040 |
| Previous ischemic stroke | 6/58 (10.3%) | 32/306 (10.5%) | 0.979 |
| Pre-morbid mRS (median [IQR]) | 0 (0 - 2) | 0 (0 - 1) | 0.002 |
| Arterial hypertension | 41/58 (70.7%) | 221/306 (72.2%) | 0.812 |
| Diabetes mellitus | 8/58 (13.8%) | 46/306 (15.0%) | 0.808 |
| Dyslipidemia | 20/58 (34.5%) | 105/306 (34.3%) | 0.980 |
| Atrial fibrillation | 22/58 (37.9%) | 85/306 (27.8%) | 0.120 |
| **Acute Assesment** |  |  |  |
| NIHSS (median [IQR]) | 12.5 (8 - 19) | 13 (8 - 18) | 0.477 |
| MAP (median [IQR]) | 104.2 (95 - 116) | 105 (93 - 120) | 0.597 |
| Blood glucose (median [IQR]) | 122 (109.5 - 152.5) | 121 (104 - 148) | 0.526 |
| ASPECTS/pc-ASPECTS (median [IQR]) | 9.5 (8 - 10) | 9 (8 - 10) | 0.914 |
| Good Collateral status | 38/58 (65.5%) | 215/306 (70.3%) | 0.472 |
| LVO (vs MeVO) | 47/58 (81.0%) | 264/306 (86.3%) | 0.300 |
| Anterior circulation | 54/58 (93.1%) | 281/306 (91.8%) | 0.743 |
| IVT | 17/58 (29.3%) | 97/306 (31.7%) | 0.719 |
| OTG (median [IQR]) | 263.5 (185 - 615) | 271 (185 - 633) | 0.794 |
| OTR (median [IQR]) | 302 (228 - 671) | 332.5 (224 - 682) | 0.855 |
| mTICI >= 2b | 51/58 (87.9%) | 276/306 (90.2%) | 0.601 |
| Any haemorrhagic transformation | 9/58 (15.5%) | 45/306 (14.7%) | 0.873 |
| Symptomatic haemorrhagic transformation | 2/58 (3.5%) | 9/306 (2.9%) | 0.690 |
| **Outcomes** |  |  |  |
| mRS at 90-day (median [IQR]) | 4 (1 - 6) | 2 (0 - 4) | 0.001 |
| mRS 0-2 at 90-day | 12/36 (33.3%) | 166/306 (54.8%) | 0.018 |
| Death at 90-day | 16/58 (27.6%) | 45/306 (14.7%) | 0.016 |

| **Supplementary Table S2**. Univariate logistic regression analysis of baseline and procedural variables associated with 90-day mRS | | |
| --- | --- | --- |
|  | OR (95% CI) | p-value |
| Age | 1.06 (1.05 - 1.08) | 0.001* |
| Female gender | 0.62 (0.41 - 0.92) | 0.019* |
| Caucasian ethnicity | 1.30 (0.36 - 4.74) | 0.694 |
| BMI | 0.96 (0.92 - 1.01) | 0.103 |
| Active smoking | 0.70 (0.42 - 1.17) | 0.168 |
| Previous ischemic stroke | 1.43 (0.75 - 2.70) | 0.277 |
| Pre-morbid mRS | 1.89 (1.54 - 2.33) | 0.001* |
| Arterial hypertension | 2.19 (1.39 - 3.44) | 0.001* |
| Diabetes mellitus | 1.62 (0.93 - 2.84) | 0.089* |
| Dyslipidemia | 0.92 (0.60 - 1.40) | 0.690 |
| Atrial fibrillation | 2.02 (1.29 - 3.15) | 0.002* |
| NIHSS | 1.13 (1.09 - 1.17) | 0.001* |
| MAP | 1.01 (1.00 - 1.02) | 0.001* |
| Blood glucose | 1.01 (1.00 - 1.01) | 0.001* |
| ASPECTS/pc-ASPECTS | 0.67 (0.58 - 0.79) | 0.001* |
| Good collateral status | 0.49 (0.35 - 0.68) | 0.001* |
| LVO (vs MeVO) | 1.06 (0.61 - 1.83) | 0.831 |
| Anterior circulation | 0.69 (0.33 - 1.42) | 0.313 |
| IVT | 0.72 (0.47 - 1.09) | 0.122 |
| OTG | 1.00 (1.00 - 1.00) | 0.089* |
| EVT Passes | 1.25 (1.06 - 1.47) | 0.008* |
| OTR | 1.00 (1.00 - 1.00) | 0.358 |
| EVT Complication | 1.79 (0.91 - 3.53) | 0.092* |
| mTICI >= 2b | 0.53 (0.28 - 1.00) | 0.050* |
| Any haemorrhagic transformation | 2.77 (1.58 - 4.84) | 0.001* |
| Symptomatic haemorrhagic transformation | 1.48 (0.45 - 4.83) | 0.514 |
| Risk of Malnutrition | 1.96 (1.31 - 2.93) | 0.001* |

| **Supplementary Table S3.** Univariate logistic regression analysis of baseline and procedural variables associated with 90-day mortality | | |
| --- | --- | --- |
|  | OR (95% CI) | p-value |
| Age | 1.06 (1.03 - 1.10) | 0.001* |
| Female gender | 0.78 (0.41 - 1.49) | 0.455 |
| Caucasian ethnicity | 1.21 (0.15 - 10.10) | 0.859 |
| BMI | 0.96 (0.90 - 1.04) | 0.304 |
| Active smoking | 0.84 (0.35 - 2.00) | 0.691 |
| Previous ischemic stroke | 1.39 (0.54 - 3.60) | 0.496 |
| Pre-morbid mRS | 1.65 (1.25 - 2.19) | 0.000* |
| Arterial hypertension | 1.94 (0.86 - 4.34) | 0.110 |
| Diabetes mellitus | 1.79 (0.81 - 3.92) | 0.148 |
| Dyslipidemia | 1.33 (0.70 - 2.55) | 0.385 |
| Atrial fibrillation | 2.16 (1.12 - 4.15) | 0.021* |
| NIHSS | 1.09 (1.03 - 1.14) | 0.002* |
| MAP | 0.99 (0.97 - 1.01) | 0.344 |
| Blood glucose | 1.01 (1.00 - 1.02) | 0.005* |
| ASPECTS/pc-ASPECTS | 0.74 (0.61 - 0.90) | 0.002* |
| Good collateral status | 0.55 (0.35 - 0.87) | 0.011* |
| LVO (vs MeVO) | 1.75 (0.59 - 5.16) | 0.313 |
| Anterior circulation | 0.66 (0.24 - 1.87) | 0.438 |
| IVT | 0.49 (0.23 - 1.07) | 0.072* |
| OTG | 1.00 (0.99 - 1.01) | 0.524 |
| EVT Passes | 1.22 (0.97 - 1.53) | 0.095* |
| OTR | 1.00 (1.00 - 1.00) | 0.935 |
| EVT Complication | 1.29 (0.46 - 3.60) | 0.630 |
| mTICI >= 2b | 0.85 (0.31 - 2.34) | 0.750 |
| Any haemorrhagic transformation | 2.16 (1.00 - 4.66) | 0.050* |
| Symptomatic haemorrhagic transformation | 1.69 (0.34 - 8.40) | 0.523 |
| Risk of Malnutrition | 2.41 (1.26 - 4.62) | 0.008* |

| **Supplementary Table S4. Association of Risk of Malnutrition (GNRI ≤ 98) and Moderate/Severe Risk of Malnutrition (GNRI < 92) With 90-Day Functional Outcome and Mortality** | | | | |
| --- | --- | --- | --- | --- |
|  | **90-day mRS** | | **90-day mortality** | |
|  | ***aOR (95%CI)**** | ***P value*** | ***aOR (95%CI)**** | ***P value*** |
| **Risk of Malnutrition+** | 1.56 (0.98 - 2.46) | 0.059 | 2.29 (1.03 - 5.10) | 0.042 |
| **Moderate/Severe Risk of Malnutrition** | 1.31 (1.00 - 1.73) | 0.054 | 1.73 (1.09 - 2.74) | 0.020 |
| * Adjusted for predefined set of prognostically relevant covariates, irrespective of their univariate statistical association. These included: age, pre-stroke mRS, diabetes mellitus and admission blood glucose, baseline NIHSS, ASPECTS, collateral status, anterior versus posterior circulation occlusion, intravenous thrombolysis, onset-to-recanalization time, successful reperfusion (mTICI ≥2b), EVT-related complications, and symptomatic intracranial hemorrhage. | | | | |
